# Severe brain atrophy after long-term survival seen in siblings with familial amyotrophic lateral sclerosis and a mutation in the *optineurin *gene: a case series

**DOI:** 10.1186/1752-1947-5-573

**Published:** 2011-12-12

**Authors:** Hiroki Ueno, Keitaro Kobatake, Masayasu Matsumoto, Hiroyuki Morino, Hirofumi Maruyama, Hideshi Kawakami

**Affiliations:** 1 Department of Clinical Neuroscience and Therapeutics, Hiroshima University, Graduate School of Biomedical Sciences, Hiroshima, Japan; 2 Department of Neurology, Kobatake Hospital, Fukuyama, Japan; 3 Department of Epidemiology, Research Institute for Radiation Biology and Medicine, Hiroshima University, Hiroshima, Japan

## Abstract

**Introduction:**

Previous studies have shown widespread multisystem degeneration in patients with sporadic amyotrophic lateral sclerosis who develop a total locked-in state and survive under mechanical ventilation for a prolonged period of time. However, the disease progressions reported in these studies were several years after disease onset. There have been no reports of long-term follow-up with brain imaging of patients with familial amyotrophic lateral sclerosis at an advanced stage of the disease. We report the cases of siblings with amyotrophic lateral sclerosis with homozygous deletions of the exon 5 mutation of the gene encoding optineurin, in whom brain computed tomography scans were followed up for more than 20 years.

**Case presentation:**

The patients were a Japanese brother and sister. The elder sister was 33 years of age at the onset of disease, which began with muscle weakness of her left lower limb. Two years later she required mechanical ventilation. She became bedridden at the age of 34, and died at the age of 57. A computed tomography scan of her brain at the age of 36 revealed no abnormality. Atrophy of her brain gradually progressed. Ten years after the onset of mechanical ventilation, atrophy of her whole brain, including the cerebral cortex, brain stem and cerebellum, markedly progressed. Her younger brother was 36 years of age at the onset of disease, which presented as muscle weakness of his left upper limb. One year later, he showed dysphagia and dysarthria, and tracheostomy ventilation was performed. He became bedridden at the age of 37 and died at the age of 55. There were no abnormal intracranial findings on brain computed tomography scans obtained at the age of 37 years. At the age of 48 years, computed tomography scans showed marked brain atrophy with ventricular dilatation. Subsequently, atrophy of the whole brain rapidly progressed as in his elder sister.

**Conclusion:**

We conclude that a homozygous deletion-type mutation in the optineurin gene may be associated with widespread multisystem degeneration in amyotrophic lateral sclerosis.

## Introduction

Previous studies have shown widespread multisystem degeneration in patients with sporadic amyotrophic lateral sclerosis (ALS) who develop a total locked-in state and survive under mechanical ventilation for a prolonged period of time [1-4]. However, the disease progressions reported in these studies were several years after disease onset. There has been no report of familial ALS patients with prolonged follow-up using brain imaging at an advanced stage of the disease. We recently reported that there are mutations in the gene encoding optineurin (*OPTN*) in patients with ALS. Here, we report siblings with ALS, with a mutation of *OPTN *in Family 1 [5], in whom follow-up with brain computed tomography (CT) scans was carried out for more than 20 years.

### Case presentation

#### Case 1

The eldest sister of three siblings in a consanguineous Japanese family developed left leg weakness at the age of 33 years. There was subsequent progression in the decrease in muscle strength accompanied by muscle atrophy in her left hand, right hand and right leg. Dysarthria and dysphagia gradually developed, and spasticity in her four limbs progressed. Neurological examination showed enhanced tendon reflexes in her four limbs and bilateral Babinski signs. Tracheostomy ventilation was performed at the age of 35 years, and complete paralysis of all four limbs was observed at the age of 40 years. By the age of 46 years, ocular movements were absent, and communication was impossible. She died of respiratory failure at the age of 57 years. An autopsy examination was not available.

**Figure 1:**
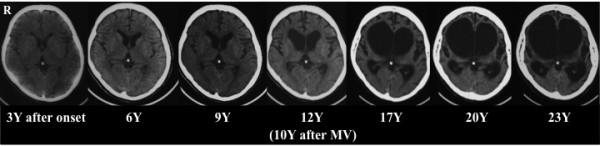
**Computed tomography scans of the older sibling**. At the age of 45 years (12 years after amyotrophic lateral sclerosis onset; 10 years after the initiation of mechanical ventilation), the scan shows marked brain atrophy. After the subsequent 10 years, whole brain atrophy had markedly progressed. Y-Years; MV-mechanical ventilation.

The total duration of the disease was 25 years. Until the age of 40 years old, when communication became impossible, there were no findings suggestive of cognitive impairment. Peripheral nerve conduction tests showed no delay in conduction, while needle electromyography revealed denervation in the active or chronic stage. Brain CT scans at the age of 36 revealed no abnormality. Atrophy of her entire brain gradually progressed, and brain CT scans at the age of 46 years old (10 years after the initiation of mechanical ventilation) showed marked ventricular dilatation. Subsequently, the atrophy of her entire brain, including her cerebral cortex, brain stem and cerebellum, markedly progressed. After another 10 years, her whole brain was markedly atrophied (Figure 1).

#### Case 2

This patient was the younger brother of Case 1. He developed a loss of left hand strength at the age of 35 years. His symptoms progressed as in his elder sister. Mechanical ventilation was initiated at the age of 38 years. At the age of 52 years, a lower gastrointestinal endoscopy, performed due to melena, showed a tumor in his descending colon. A biopsy demonstrated adenocarcinoma. At the age of 55 years, black feces were observed, and an upper gastrointestinal endoscopy showed a submucosal tumor on the lesser curvature of his stomach. A biopsy demonstrated papillary adenocarcinoma. He died of gastric cancer in the same year. An autopsy examination was not available.

**Figure 2:**
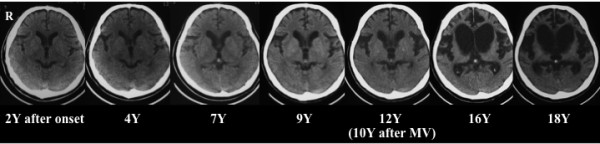
**Computed tomography scans of the younger sibling**. At the age of 48 years (12 years after onset; 10 years after the initiation of mechanical ventilation) brain atrophy was apparent. Subsequently, brain atrophy progressed as in his elder sister. Y-Years; MV-mechanical ventilation.

The entire duration of the disease was 21 years. Until the age of 38 years, when communication became impossible, no clear cognitive impairment was observed. Nerve conduction tests revealed no findings suggesting peripheral neuropathy, while needle electromyography showed neurogenic changes. On CT scans obtained at the age of 37 years, there were no abnormal intracranial findings. At the age of 48 years (10 years after the initiation of mechanical ventilation), CT scans showed marked ventricular dilatation. Subsequently, atrophy of his whole brain rapidly progressed as in his elder sister. CT scans at the age of 54 years showed severe atrophy of his cerebral cortex and cerebral white matter accompanied by marked ventricular dilatation (Figure 2).

The younger sister was unaffected.

## Discussion

There is controversy as to whether ALS affects only motor neurons. In our cases, there was marked atrophy of both the cortex and white matter about 20 years after disease onset, suggesting extensive neuronal loss and gliosis beyond the motor system. Brain atrophy appeared to accelerate 10 years after the initiation of mechanical ventilation. According to previous reports, a neuropathological feature of ALS with long-term survival is widespread multisystem degeneration with obvious neuronal loss [1-4]. However, since autopsies could not be performed in either patient, detailed pathological evidence could not be obtained. In addition, based on the pattern of ventricular dilatation, hydrocephalus may have been involved. A change of intracranial pressure due to the long-term confinement to bed (about 20 years) may have been associated with the circulatory failure of cerebrospinal fluid.

A recent review indicates that ALS is a multisystem disorder rather than a pure lower and/or upper motor neuron disorder [6]. However, not all ALS patients show the widespread multisystem degeneration observed in long-term survivors under mechanical ventilation, and the reason for this difference is unclear [7,8]. Nishihira *et al*. [2] reported widespread multisystem degeneration in a patient with sporadic ALS who had survived for long periods with mechanical ventilation. Their report showed, on immunohistochemistry, that TAR DNA-binding protein of 43 kDa (TDP-43)-immunoreactive and ubiquitin-immunoreactive cytoplasmic inclusions was widespread in the nervous system, including the motor and non-motor neuron systems. TDP-43 was identified as a major disease protein in ubiquitinated inclusions in sporadic and familial frontotemporal lobar degeneration with ubiquitin-positive inclusions (FTLD-U) and sporadic ALS [9,10]. The siblings' brain atrophy pattern in our study was similar to FTLD in morphology. We demonstrated that OPTN is co-localized with TDP-43 in the pathognomonic inclusion bodies of sporadic ALS [5]. It was concluded that the widespread multisystem degeneration in ALS with the *OPTN *mutation may be associated with FTLD-U.

Ito *et al*. [11] investigated Family 4 [5] with a heterozygous E478G missense type *OPTN *mutation, and reported that neuroimaging showed medial temporal lobe atrophy. The pathomechanism causing the disease may be different dependent on a recessive and dominant mutations; ALS due to a recessive mutation is speculated to show a loss of function resulting from nonsense-mediated mRNA decay of transcription [5]. The difference in patterns of brain atrophy between recessive and dominant traits may reflect different pathomechanisms.

## Conclusion

This report is, to our knowledge, the first to show prolonged follow-up with brain imaging of familial ALS patients at an advanced stage of the disease. The siblings in this study had an exon 5 homozygous deletion-type *OPTN *mutation, suggesting the association between an *OPTN *mutation-type and the presence of such widespread degeneration.

## Consent

Written informed consent was obtained from the husband of the elder sister and the wife of the younger brother for publication of this case series and any accompanying images. Copies of the written consent are available for review by the Editor-in-Chief of this journal.

## Competing interests

The authors declare that they have no competing interests.

## Authors' contributions

HU compiled the data and interpreted the CT studies. KK analyzed and interpreted the patient data regarding the neurological disease. HMa, HMo and HK diagnosed the OPTN mutation. MM, HM, HM and HK helped conceive the study and design and helped co-ordinate the study. HU was a major contributor in writing the manuscript. All authors read and approved the final manuscript.
